# Keratinized Mucosa Width and Peri‐Implant Health: Clinical Associations to Inform Soft Tissue Evaluation

**DOI:** 10.1155/ijod/5248770

**Published:** 2026-07-20

**Authors:** Kavitha Parthasarathy, Josef Huang, Didem Ozdemir Ozenen, Navid Knight

**Affiliations:** ^1^ Department of Periodontics, University of the Pacific, Arthur A. Dugoni School of Dentistry, San Francisco, California, USA, pacific.edu; ^2^ Associate Dean for Oral Health Education, University of the Pacific, Arthur A. Dugoni School of Dentistry, San Francisco, California, USA, pacific.edu

**Keywords:** keratinized mucosa, mucosal recession, peri-implant health, soft tissue augmentation

## Abstract

**Objective:**

The clinical necessity of keratinized mucosa (KM) around dental implants remains debated. This study examines whether KM width influences peri‐implant soft tissue health and informs decisions for soft tissue augmentation.

**Methods:**

In this cross‐sectional study, 56 adults with 101 dental implants in function for at least 12 months were examined. Implants were categorized by midfacial KM width as ≥2 mm (*n* = 50) or <2 mm (*n* = 51). Clinical parameters included plaque index (PI), bleeding on probing (BOP), probing depth (PD), and mucosal recession (MR), analyzed using mixed‐effects models and generalized estimating equations (GEE) to account for multiple implants per patient.

**Results:**

After accounting for patient‐level clustering, implants with <2 mm KM demonstrated higher BOP (45.1% vs. 26.0%; OR = 0.39, 95% CI [0.18–0.88], *p* = 0.023), greater plaque accumulation (0.90 vs. 0.56; *β* = −0.29, 95% CI [−0.53, −0.05], *p* = 0.019), and increased MR (0.12 mm vs. 0.00 mm; *β* = −0.12, 95% CI [−0.21, −0.03], *p* = 0.009) compared with those having ≥2 mm KM. No significant differences in PD were observed.

**Conclusion:**

A KM width of at least 2 mm is associated with reduced peri‐implant inflammation and recession. These findings suggest that reduced KM width may be associated with adverse peri‐implant parameters and could serve as a clinical consideration during assessment.

**Practical Implications:**

Adequate KM may reduce peri‐implant inflammation and recession. Evaluating KM width during periodontal maintenance visits may help identify sites warranting further soft tissue evaluation by a periodontist.

## 1. Introduction

Implant‐supported restorations have become a cornerstone of contemporary tooth replacement, providing reliable functional and esthetic rehabilitation across a broad range of clinical presentations. With their expanding role in everyday dental practice, the sustained health of the surrounding peri‐implant tissues has emerged as a defining determinant of long‐term treatment success [1]. While osseointegration is essential for implant survival, the integrity and health of peri‐implant soft tissues play a central role in maintaining long‐term stability of the implants, serving as a biological barrier against microbial invasion and mechanical trauma, and contributing to both functional and esthetic outcomes [2, 3].

Keratinized mucosa (KM), a dense, keratinized band of tissue surrounding natural teeth and implants, has been proposed to support peri‐implant tissue health through several mechanisms. Clinically, KM is thought to resist mechanical trauma during mastication and oral hygiene, enhance patient comfort while brushing, and facilitate plaque control by providing a stable, protective tissue interface [3–7]. Multiple studies have suggested that a width of ≥2 mm of KM is associated with improved peri‐implant soft tissue outcomes, including lower plaque accumulation, reduced bleeding on probing (BOP), shallower probing depths (PDs), and diminished mucosal recession (MR) [2, 4, 6].

Despite evidence supporting the role of KM in reducing inflammation, MR, and patient discomfort, current clinical protocols primarily focus on implant osseointegration and prosthetic design, with less emphasis on assessing or optimizing the peri‐implant mucosal phenotype throughout the continuum of care. Uncertainty about when to refer patients for soft tissue augmentation around implants poses a significant challenge for general dentists. Clinical indicators such as BOP, plaque accumulation, MR, and patient‐reported discomfort are commonly used to guide decisions, yet evidence‐based thresholds for intervention have not been well established.

The present study addresses these gaps by providing practice‐based clinical data from a standardized maintenance population, offering a patient‐level analysis that accounts for clustering of multiple implants within the same individual, a methodological consideration largely absent from prior cross‐sectional studies in this area. Identifying clinical associations with KM width may help translate research findings into practical guidance and support long‐term peri‐implant health.

## 2. Methods

This cross‐sectional clinical study included 56 adult patients with a total of 101 dental implants that had been in function for at least 12 months. Review of available clinical records demonstrated that the functional duration of implants ranged from ~1 to 12 years among cases for which documentation was available. All examinations were conducted at the Arthur A. Dugoni, University of the Pacific School of Dentistry following Institutional Review Board approval (IRB2025‐126), and informed consent was obtained from all participants.

Patients were assigned to one of two groups based on the width of KM at the midfacial aspect of the implant: Group A included implants with ≥2 mm of KM (*n* = 50) and Group B included implants with <2 mm or no KM (*n* = 51). Inclusion criteria were age ≥18 years and the presence of at least one implant in function for ≥12 months. Patients were excluded if they had active periodontal disease, a history of soft tissue grafting at the implant site, uncontrolled diabetes mellitus, or were current smokers. All patients were enrolled in a routine maintenance program and received professional prophylaxis every 6 months, ensuring standardized supportive care.

Prior to data collection, all examiners underwent calibration using volunteer patients not included in the study. Each examiner measured PD, MR, and KM at multiple implant sites. Calibration sessions were continued until examiners achieved consistent agreement within ±1 mm. While formal inter or intraexaminer reliability metrics (e. g., intraclass correlation coefficient [ICC] or kappa) were not calculated, agreement was assessed clinically through consensus and verification against a reference examiner.

Clinical measurements were performed using a Marquis periodontal probe (Hu‐Friedy, Chicago, IL, USA) for KM and MR and a plastic periodontal probe (PCP‐UNC 15, Hu‐Friedy, Chicago, IL, USA) for PD. Clinical parameters were recorded during routine periodontal exams and prophylaxis visits. KM and MR were measured at the midfacial aspect, while PD was measured at six sites per implant using a plastic probe. Plaque accumulation was assessed using the Löe and Silness Plaque Index (PI) (0–3), where 0 = no plaque, 1 = thin film detectable only with a probe or disclosing solution, 2 = moderate visible deposits, and 3 = abundant plaque within the gingival pocket and/or on the tooth and gingival margin. BOP was recorded dichotomously (present/absent) at six sites per implant. All findings were recorded on standardized forms and entered into a secure, deidentified electronic database.

Power analysis indicated that a total sample size of 80 implants (40 per group) was sufficient to detect a moderate effect size (Cohen’s *d* ≈ 0.50) with 80% power at *α* = 0.05 based on differences in plaque accumulation and BOP between groups.

Descriptive statistics were computed for all variables. In addition to analyzing the six individual PD measurements, the mean PD across all six sites was computed per implant. Because 56 patients contributed a total of 101 implants (range: 1–5 implants per patient), with 15 patients contributing implants to both KM groups, standard tests that assume the independence of observations were not appropriate. To account for within‐patient clustering, all primary analyses used mixed‐effects models. Continuous outcomes (PI, mean PD, and MR) were analyzed using linear mixed‐effects models (LMM) with the patient as a random intercept (REML estimation). BOP, a binary outcome, was analyzed using generalized estimating equations (GEE) with a binomial family, logit link, and exchangeable within‐patient correlation structure with robust sandwich standard errors. ICCs were computed from the LMM variance components to quantify the degree of clustering for each outcome. Effect sizes are reported as the fixed‐effect coefficient *β* (continuous outcomes) and the odds ratio (BOP). A *p*‐value of <0.05 was considered statistically significant. Prior to LMM analysis, the normality of residuals was assessed via visual inspection of Q–Q plots; assumptions were considered adequately met given the robustness of mixed‐effects models to moderate violations of normality.

## 3. Results

A total of 101 implants from 56 unique patients were analyzed, with 51 implants in the <2 mm KM group (26 patients) and 50 implants in the ≥2 mm KM group (30 patients); 15 patients contributed implants to both groups. ICCs computed from the LMM variance components confirmed meaningful within‐patient clustering for PI (ICC = 0.63) and mean PD (ICC = 0.56) and moderate clustering for recession (ICC = 0.24) and BOP (ICC = 0.23), justifying the use of mixed‐effects models as the primary analytic approach. Descriptive statistics and clustering‐adjusted inferential results are summarized in Table 1.

**Table 1:** Descriptive statistics and clustering‐adjusted group comparisons for clinical outcomes.

Outcome	<2 mm (*n* = 51)	≥2 mm (*n* = 50)	Model and test statistic	*p*	Effect size/ICC
BOP, *n* (%)	23 (45.1)	13 (26.0)	GEE (binomial, exchangeable)	0.023	OR = 0.39 [0.18–0.88]
Recession	M = 0.12 (0.33)	M = 0.00 (0.00)	LMM; *β* = −0.12 [−0.21, −0.03]	0.009	ICC = 0.24
Plaque index	M = 0.90 (0.88)	M = 0.56 (0.64)	LMM; *β* = −0.29 [−0.53, −0.05]	0.019	ICC = 0.63
Mean PD	M = 3.12 (0.86)	M = 3.18 (0.65)	LMM; *β* = −0.04 [−0.30, 0.22]	0.788	ICC = 0.56

*Note:* Values in parentheses are standard deviations. LMM = linear mixed‐effects model with random intercept per patient (REML); GEE = generalized estimating equations (binomial, exchangeable correlation, robust SE); ICC = intraclass correlation coefficient from LMM variance components; *β* = fixed‐effect coefficient for group (≥2 mm vs. <2 mm). Mean PD = mean of six probing depth sites per implant.

Abbreviations: BOP, bleeding on probing; OR, odds ratio.

### 3.1. BOP

**Figure 1:**
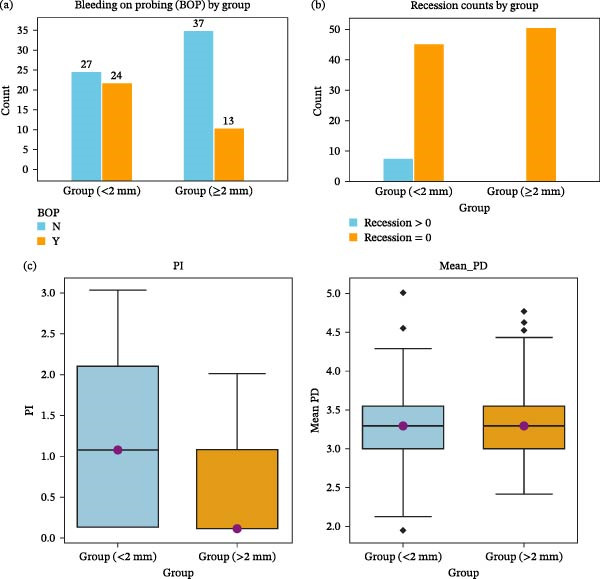
Distribution of clinical outcomes by keratinized mucosa group. (a) Percentage of implants exhibiting bleeding on probing (BOP) in the <2 mm and ≥2 mm of KM groups. (b) Mucosal recession values for implants in the <2 mm and ≥2 mm of KM groups. (c) Plaque index (PI) and mean probing depth (PD) values for implants in the <2 mm and ≥2 mm of KM groups. Bar charts display the percentage of implants with BOP and the mean recession values in each group. Boxplots illustrate the distribution of PI and mean PD values. In the boxplots, the central line represents the median, the box represents the interquartile range, whiskers indicate the range of nonoutlier values, and outliers are displayed as individual points.

GEE analysis indicated that implants in the <2 mm group were more likely to exhibit BOP (45.1%) than those in the ≥2 mm group (26.0%; OR = 0.39 95% CI [0.18–0.88], *p* = 0.023). This corresponds to implants with ≥2 mm KM having significantly lower odds of presenting with BOP (61% reduction in odds) (Figure 1a).

### 3.2. Recession

Recession was significantly greater in the <2 mm group (M = 0.12 mm, SD = 0.33) compared with the ≥2 mm group (M = 0.00 mm, SD = 0.00), with a fixed‐effect coefficient of *β* = −0.12 (95% CI [−0.21, −0.03], *p* = 0.009) (Figure 1b).

### 3.3. PI

Mean PI scores were significantly higher in the <2 mm group (M = 0.90, SD = 0.88) than in the ≥2 mm group (M = 0.56, SD = 0.64), with a fixed‐effect coefficient of *β* = −0.29 (95% CI [−0.53, −0.05], *p* = 0.019) (Figure 1c).

### 3.4. PDs

Mean PD was 3.12 mm (SD = 0.86) in the <2 mm group and 3.18 mm (SD = 0.65) in the ≥2 mm group. No statistically significant difference was observed between groups (*β* = −0.04, 95% CI [−0.30, 0.22], *p* = 0.788) (Figure 1c).

## 4. Discussion

This study reinforces the importance of KM in maintaining peri‐implant soft tissue health. Consistent with previous studies [1–6], implants with <2 mm of KM exhibited higher plaque accumulation, increased BOP, and greater MR than implants with ≥2 mm of KM. Importantly, all patients in our study were enrolled in a standardized 6‐month prophylaxis recall program, minimizing variability in professional maintenance care and strengthening the likelihood that the observed differences in plaque, BOP, and recession are related to KM width rather than irregular maintenance.

Although several findings reached statistical significance after accounting for patient‐level clustering, the clinical magnitude of some differences warrants careful interpretation. The mean difference in MR between groups was 0.12 mm, which, while statistically significant, may be of limited standalone clinical significance. Similarly, no significant differences were observed in PD. PI and BOP differences, though statistically significant, reflect small‐to‐moderate effect sizes (*β* = −0.29 and OR = 0.39, respectively). Collectively, these findings support an association between KM width <2 mm and less favorable peri‐implant soft tissue parameters. While KM is not strictly required for implant survival, these findings indicate that it contributes meaningfully to peri‐implant stability, patient comfort, and effective plaque control [2–4, 6–8].

Radiographic bone‐level analysis was not included in this study because the primary focus was peri‐implant soft tissue parameters. In a cross‐sectional assessment, interpretation of isolated radiographic bone levels may be challenging without standardized baseline radiographs as physiologic remodeling cannot always be distinguished from progressive peri‐implant bone loss. Previous studies have also not established a consistent association between KM width and marginal bone loss [7, 9]. Nevertheless, the absence of radiographic evaluation limits the comprehensive assessment of peri‐implant health and should be considered when interpreting the findings.

No significant differences were observed in PDs between groups, aligning with prior reviews that show KM width has a stronger relationship with plaque, BOP, and MR than with pocket depth [4, 10, 11]. The absence of PD differences in this study may reflect the cross‐sectional design, which captures the peri‐implant status at a single point in time, as well as the inclusion of systemically and periodontally healthy patients, reducing the likelihood of detecting PD changes. Progressive changes or outcomes in higher‐risk populations may differ, emphasizing the need for longitudinal studies to determine whether <2 mm KM contributes to increased PDs or peri‐implant disease over time. The lack of differences in PD can also be attributed to the fact that greater recession results in shallower PD, as reported in other studies [10].

The 2023 AAP/AO Consensus Workshop highlighted the importance of the peri‐implant soft tissue phenotype, including KM width and mucosal thickness, identifying inadequate KM or thin mucosa as site‐level risk indicators for recession and peri‐implant defects [12]. Our findings quantitatively support this consensus by demonstrating that implants with <2 mm of KM are associated with higher plaque, BOP, and recession, providing empirical evidence for the clinical relevance of KM assessment. While the association between reduced KM and adverse peri‐implant parameters has been reported previously, the present study adds to this evidence base by demonstrating that these associations persist after accounting for within‐patient clustering, a methodological consideration absent from many prior cross‐sectional studies, and does so in a population receiving standardized maintenance care. Beyond clinical measures, KM also influences patient‐centered outcomes. Prior research indicates that adequate KM improves comfort during oral hygiene, facilitates plaque control, and enhances long‐term satisfaction, whereas insufficient KM can cause brushing discomfort, discouraging effective oral hygiene and increasing the risk of inflammation and MR [13]. By routinely assessing KM, clinicians can identify sites that may warrant treatment early and integrate both clinical and patient‐centered considerations into intervention decisions.

However, it is important to note that the relationship between KM width and peri‐implant clinical outcomes is not uniformly supported in the literature. Some studies have reported minimal or no significant association between KM width and PD or marginal bone levels, suggesting that adequate oral hygiene and maintenance may mitigate the influence of KM in certain patient populations. These variations across studies may stem from differences in methodology, patient populations, duration of follow‐up, and the criteria used to characterize peri‐implant health outcomes [14, 15].

Soft tissue augmentation has been explored as both a preventive and therapeutic approach at implant sites with limited KM. Free gingival grafts (FGG), connective tissue grafts (CTG), and xenogeneic collagen matrices (XCM) have been shown to effectively increase KM and improve peri‐implant tissue stability. Systematic reviews indicate that CTG generally provides greater gains in tissue thickness and superior clinical and esthetic outcomes compared with XCM, although XCM remains a viable alternative when autogenous tissue is limited [16–18]. Long‐term evidence demonstrates that augmentation maintains stable mucosal margins, KM width, and marginal bone levels, supporting the durability and effectiveness of these procedures. Patient‐reported outcomes further suggest that augmented KM improves comfort during oral hygiene and facilitates long‐term maintenance [13, 19–21]. Decisions regarding augmentation should also consider implant positioning and prosthetic factors. When tissue deficiency arises from suboptimal implant positioning or prosthetic design, modification or replacement should be considered prior to grafting [22].

Overall, these findings indicate that <2 mm KM is associated with increased risk of peri‐implant inflammation and recession. KM width may therefore represent a clinically relevant indicator to consider when evaluating the potential need for soft tissue assessment while ensuring optimal implant placement and prosthetic design, though longitudinal data are required to establish predictive and causal relationships.

## 5. Conclusion

KM around dental implants is associated with peri‐implant soft tissue health. This study demonstrates that implants with <2 mm of KM are associated with significantly higher plaque accumulation, increased BOP, and greater MR compared with those with ≥2 mm of KM. While cross‐sectional design precludes causal inference, these associations suggest that KM width may serve as a clinically relevant indicator for identifying sites that warrant further soft tissue evaluation.

These associations may inform clinical assessment in the following ways:

• ≥2 mm KM with healthy peri‐implant tissues: continue routine maintenance and monitoring.
• <2 mm KM without clinical signs: these sites may benefit from closer monitoring and soft tissue evaluation by a periodontist.
• <2 mm KM with active clinical signs (BOP, progressive recession, and patient discomfort): these sites may indicate increased risk and should prompt clinicians to consider management of peri‐implant mucositis and referral for soft tissue evaluation and augmentation to prevent progression to peri‐implantitis.

The associations identified in this study are consistent with existing literature and provide a foundation for clinical awareness, though longitudinal and interventional studies are needed to confirm whether these associations are predictive and to establish evidence‐based intervention thresholds so that early identification of insufficient KM can inform timely clinical assessment and decision making.

## 6. Limitations

This study has limitations that should be considered when interpreting the findings. The cross‐sectional design precludes causal inference and does not allow the assessment of longitudinal changes in peri‐implant tissues over time. Baseline radiographic data were not consistently available, and bone‐level parameters were therefore excluded as outcomes. Implant functional duration was not uniformly available for all cases.

The study population was drawn exclusively from patients enrolled in a structured maintenance program, which, while ensuring consistent supportive care, may limit generalizability to higher‐risk populations. Soft tissue phenotype and oral hygiene practices were not assessed. With an effective patient‐level sample of 56 individuals, simultaneous adjustment for multiple covariates risks overfitting; formal multivariable analysis is better suited to a prospectively designed study with a larger sample and prespecified covariate collection.

Residual confounding from implant‐level variables and patient‐related variables could not be excluded and may have influenced the observed associations.

Finally, formal inter and intraexaminer reliability statistics were not calculated. All examiners underwent structured calibration against a reference examiner prior to data collection, with agreement verified within ±1 mm, consistent with the methodology reported in comparable peri‐implant studies. Prospective reporting of ICC or kappa values would strengthen the reliability assessment in future work.

## 7. Future Directions

Future prospective longitudinal studies are needed to determine whether KM width <2 mm has predictive value for progressive peri‐implant tissue changes or disease development and whether soft tissue augmentation at these sites modifies clinical outcomes. Research should also extend to higher‐risk populations, including patients with periodontitis, uncontrolled systemic conditions, or inconsistent maintenance, among whom the clinical impact of inadequate KM may be more pronounced.

## Funding

This study was supported by the University of the Pacific, Arthur A. Dugoni School of Dentistry, Research Pilot Project Award D30060‐Activity 161.

## Conflicts of Interest

The authors declare no conflicts of interest.

## Data Availability

The data that support the findings of this study are available upon request from the corresponding author. The data are not publicly available due to privacy or ethical restrictions.
